# Local Examination and Treatment of Disease of the Upper Rectum Sigmoid Flexure

**Published:** 1898-10

**Authors:** 


### LOCAL EXAMINATION AND TREATMENT OF DISEASE OF THE UPPER RECTUM SIGMOID FLEXURE.

Dr. James P. Tuttle contributes a valuable article to the *New York Medical Journal*, July 2, on this subject, in which he strongly emphasizes the utility of the proctoscope or "Kelly tubes" in the diagnosis of diseases of these parts. Neoplasms, while not the most frequent lesions, are of paramount importance from a diagnostic point of view. Especially is this true of carcinoma for the reason that when neglected its cure soon becomes hopeless. The early symptoms of intestinal tumors are not only obscure but strikingly similar, and are often mistaken for functional or inflammatory disorders. But the diagnosis can be easily cleared up by the use of the proctoscope if the lesion exists within the lower twenty to thirty-five inches of the intestinal tract.

The serious forms of benign neoplasms may usually be dealt with through the tube itself by the use of proper instruments.

In treating of the non-surgical diseases mention is made of three forms of catarrh, viz., *simple, acute, hypertrophic* and *atrophic*. The latter is a very common affliction, comprising about twenty-five per cent. of the cases seen at the author's clinics. All these forms of catarrh may be recognized through the tubes as well as the several varieties of ulceration. The treatment of this class of affections consists in keeping the parts clean by means of saline or boric acid irrigations and applying the proper medication directly to the part through the Wales bougie.

The concluding paragraphs of the article are as follows:

"In conclusion, I wish to make two applications of the knowledge gained from a very large number of these local examinations of the sigmoid flexure and upper rectum.

"*Morning Diarrhea.*—The so-called morning diarrheas, the symptoms of which have been so admirably described by Dr. Francis Delafield, are, in my opinion, always due to some organic disease of the rectum, sigmoid, or descending colon. It may be cancer, polypus, papilloma, ulceration, or catarrhal disorder, but in every case I have seen I have been able to find some irritating condition of these parts to account for the diarrhea, and still more, the diarrhea has disappeared when the cause has been removed. The history of the case, the symptoms, the nature of the stool, etc., may lead us to fairly correct conclusions as to the cause of this condition, but we can never be certain until we have actually seen the parts, and upon this certainty will depend the accuracy and efficiency of our treatment.

"*Mucous Diarrhea.*—Mucous diarrhea is another condition due to actual disease. It is always associated with the follicular inflammation of the sigmoid flexure and colon. I have seen it frequently in atrophic catarrh of these parts, and perhaps other diseases may cause it, but I have not observed them. The colicky pains and exhaustion after stool accompanying this condition are due to temporary partial obstruction and consequent spasm. Prolapse or intussuception of the sigmoid is one of the most frequent causes of this. I have frequently seen this condition through the tube, and when the parts have been restored and a full high enema given, the mucus was passed without any pain at all.

"The nervous conditions associated with this form of diarrhea appear to me to be all due to the disease causing it, and not vice versa. The doctrine that it is a neurosis appears to me utterly without foundation and not tenable in view of the conditions always found."

